# NMDA receptor inhibition prevents intracellular sodium elevations in human olfactory neuroepithelial precursors derived from bipolar patients

**DOI:** 10.1038/s41598-022-14187-w

**Published:** 2022-06-21

**Authors:** Yonglin Gao, Aaron A. Mack, Carleigh Litteral, Nicholas A. Delamere, Rif S. El-Mallakh

**Affiliations:** 1 Department of Psychiatry and Behavioral Sciences, Mood Disorders Research Program, Depression Center, University of Louisville School of Medicine, Louisville, KY USA; 2 Department of Physiology, University of Arizona, PO Box 245051, Tucson, AZ 85724-5051 USA

**Keywords:** Preclinical research, Neurophysiology

## Abstract

Dysregulation of ion flux across membranes and glutamate-induced excitotoxicity appear to be important pathophysiologic abnormalities in bipolar illness. Understanding ion control and responses to ionic stress is important to decipher the pathogenesis of this disorder. Monensin alone significantly increased [Na]_i_ in ONPs from bipolar individuals (5.08 ± 0.71 vs baseline 3.13 ± 0.93, *P* = 0.03) and AP5 had no effect (2.0 ± 1.2 vs baseline 3.13 ± 0.93, P = 0.27). However, the combination of AP5 and monensin resulted in normalization of [Na]_i_ (3.25 ± 1.28 vs baseline 3.13 ± 0.93, *P* = 0.89). This effect was not observed in cells from non-bipolar individuals (monensin alone, 1.72 ± 1.10 vs baseline 2.42 ± 1.80, *P* = 0.25; AP5 alone, 1.37 ± 0.74 vs baseline 2.42 ± 1.80; AP5 combined with monensin, 1.53 ± 0.98 vs baseline 2.42 ± 1.80,* P* = 0.31). Sodium regulation is central to neuronal function and may be disturbed in patients with bipolar disorder. Monensin is an ionophore, meaning that it incorporates itself into the membrane and allows sodium to enter independent of cellular membrane proteins. While the mechanism remains obscure, the observation that the NMDA receptor antagonist, AP5, normalizes [Na]_i_ only in olfactory neuroepithelial precursors obtained from bipolar illness may provide novel insights into ion regulation in tissues from subjects with bipolar illness.

## Introduction

Bipolar disorder (BD) is a severe mental illness that afflicts about 1–3% of the population^1,2^, or about 3 to 5 million people in United States^3^. It has suboptimal outcomes regarding both disease and its treatment^4,5^. Better understanding of pathophysiologic processes would improve treatment development.

Neuroelectrical genes account for some 74% of all known susceptibility loci^6^. Furthermore, ion regulatory abnormalities are among the most reproducible findings in studies of BD^7^. Most important is the elevation of intracellular sodium ([Na]_i_) and dysfunction of ion transport systems^7,8^. Several ion regulatory systems have been shown to be uniquely disrupted in bipolar patients versus controls^7–10^. Furthermore, bipolar patients have been found to have excessive levels of the precursor glutamine in certain parts of their brain, as well as higher numbers of glutamate receptors^9–13^. These findings imply an abnormality of glutamate signaling in bipolar.

The aim of the present study was to further examine the role of the ionotropic N-methyl-D-aspartate (NMDA) glutamate receptor in the regulation of [Na]_i_. We utilized a sodium ionophore, monensin, to increase [Na]_i_ to bypass other cellular proteins and focus on NMDA receptor function.

## Methods

The olfactory neuroepithelial precursor (ONP) cells used have been previously described (BD n = 3; nonBD n = 6)^14^. Briefly, the BP subjects were a lithium-responsive 22 year-old male (on lithium for 5 years), a lithium-responsive 47 year-old male (on divalproex for 22 years), and a 56 year-old woman who had never received lithium (on divalproex for 34 years)^14^. Controls were age and gender matched. ONP cells are known to have functional NMDA receptors^14–16^. We used cells that were at passage number 15–20. ONPs were treated with the sodium ionophore monensin at 1 µM to model the elevation of [Na]_i_ present in bipolar patients. The dose and duration of treatment was based on previous work that specifically modelled the doubling of intracellular sodium concentrations seen in manic bipolar patients^7^. Specifically, treatment with 0.1 M glutamate for 6 h^16^, or 1 µM of monensin for 2 h^17^ double the [Na]_i_ in ONPs. The NMDA receptor antagonist AP5 (D-2-amino-5-phosphonopentanoate) was used to examine the role of NMDA receptor in normalizing this non-glutamate ionic stress, and was used as a pretreatment.

ONPs, which were initially obtained by olfactory epithelial biopsy after the subjects signed an informed consent; we followed all of the guidelines and regulations for human studies that is outlined in the Declaration of Helsinki and were overseen by our institutional review board (the University of Louisville Human Subjects Protection Program Office [HSPPO], protocol number 485.04). The cells were frozen in liquid nitrogen and for the experiments they were cultured in minimum essential media (MEM [content of media may be found at https://www.thermofisher.com/us/en/home/technical-resources/media-formulation.201.html; importantly, the sodium concentration is 0.84% which is similar to saline and has 2 mM L-glutamine but no glutamate]), gentamycin 0.1 mg/mL, and FBS 10%, in 5% CO_2_ as previously described^14^. They were treated with 1 µM monensin for 6.5 h, 0.1 mM AP5 for 6.5 h, or pretreated AP5 30 min followed by adding monensin to AP5 for an additional 6 h. This latter point was, in part, to ensure that depolarization with monensin does not activate the NMDA receptor. Each experiment was repeated 3–5 times. [Na]_I_, obtained in triplicate for each experiment, was measured with flame spectroscopy and expressed as concentration per protein as measured by Lowry methodology ([Na]_i_ × 10^–5^ M/mg protein). Statistical analysis employed unpaired, 2-tailed t-tests. Since sodium concentration was the only outcome measure, and only 10 comparisons made, multiple-comparison corrections were not necessary.

### Ethics approval and consents

The ONP cells were obtained by biopsy over two decades ago under a grant from the Brain and Behavior Research Foundation. At that time, all participants provided informed consent and the protocol was approved by the institution’s Human Subjects Protection Program.

## Results

**Figure 1:**
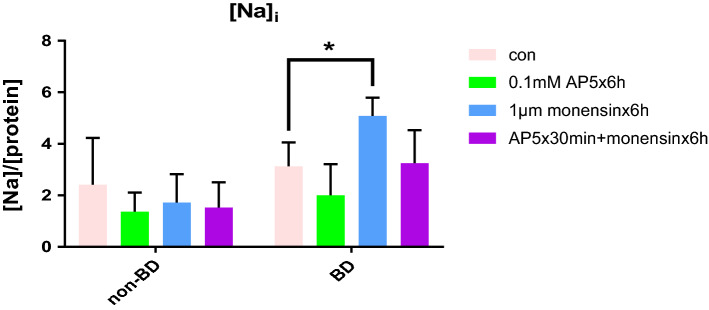
Intracellular sodium concentration with monensin treatment significantly increases only in ONPs from BD patients, but not non-bipolar controls, treated with monensin 1 µm for 6 h (*P < 0.05). BD ONPs pretreated by AP5 for 30 min followed by monensin for 6 h treatment were protected from the increase of intracellular sodium concentration.

Monensin alone significantly increased [Na]_i_ in ONPs from bipolar individuals (5.08 ± SD 0.71 vs baseline 3.13 ± 0.93 10^−5^ M/mg protein , *P* = 0.03) and AP5 alone had no effect (2.0 ± 1.2 vs baseline 3.13 ± 0.93, *P* = 0.27) (Fig. 1). The combination of AP5 and monensin normalized [Na]_i_ (3.25 ± 1.28 vs baseline 3.13 ± 0.93, *P* = 0.89). This effect was not observed in cells from non-bipolar individuals (monensin alone, 1.72 ± 1.10 vs baseline 2.42 ± 1.80, *P* = 0.25; AP5 alone, 1.37 ± 0.74 vs baseline 2.42 ± 1.80; AP5 + monensin, 1.53 ± 0.98 vs baseline 2.42 ± 1.80, *P* = 0.31) (Fig. 1).

## Discussion

ONPs derived from BD individuals appear to be more susceptible to [Na]_i_ elevations induced by monensin than nonBD controls. Despite the absence of glutamate in the experimental system, when the NMDA glutamate receptors are blocked, elevations of [Na]_i_ are blocked. NonBD cells appear to be able to compensate for monensin-induced elevations of [Na]_i_ in an NMDA receptor-independent manner. In this model, NMDA receptor appears necessary for bipolar-specific sodium ion dysregulation.

We utilized monensin, a sodium ionophore, to model the doubling of the intracellular sodium concentrations that have been documented in living humans with active mania^18,19^. Monensin can regulate the activity of NMDA receptor sodium channel^20,21^, and has been show to increase sodium pump activity^22,23^, regulate transport of sodium, potassium and calcium, and stimulate sodium-proton exchanger activity^24^. These changes appear to result from the elevation of intracellular sodium induced by monensin and have been demonstrated in a wide variety of cell types. The current study focused on the NMDA receptor, and we did not investigate other compensatory mechanisms in our ONP cells.

We did demonstrate a bipolar-specific abnormality in ion regulation, but have not determined the specific nature of the anomaly. We utilized an experimental dose that doubled the intracellular sodium concentration in cells obtained from bipolar patients. That same concentration did not increase sodium in control ONPs. We do not know if the lack of increase is related to a unique sensitivity to monensin in ONPs from bipolar individuals, or other unidentified compensatory mechanisms in ONPs from controls. However, at this concentration of monensin, an active NMDA receptor appears to play an important role in mediating the observed elevations of intracellular sodium in cells with the genetic heritage of bipolar illness (Fig. 1).

The use of a small number of diagnosis-specific cell lines is a limitation to this study, but is an acceptable practice given the labor-intensive nature of these investigations^25^. Replication of these findings in other cellular models would be important.

Sodium ion regulation appears to be altered in BD. Several systems have been implicated including the sodium pump^26^, cytoskeletal proteins^27^, and NMDA receptors^28^. The current study suggests that when the NMDA receptor is rendered dysfunctional with AP5, non-glutamate-mediated [Na]_i_ elevation is prevented. In nonBD ONPs the dose of monensin used does not increase [Na]_i_ even in the absence of NMDA dysfunction (Fig. 1). This suggests a central role of the NMDA receptor, and might explain why some NMDA antagonists, such as lamotrigine, memantine, and ketamine, are effective in the treatment of bipolar disorder.

We have previously demonstrated at 0.1 M glutamate treated for 6 h increased intracellular sodium ([Na]_i_) concentration twice that of untreated ONPs^9^. Similarly, ONPs derived from BD were more susceptible to glutamate-induced apoptosis^17^. The diagnosis-specific abnormalities related to ion regulation, along with the unique effectiveness of medications that alter ion function in bipolar disorder^29^ support ongoing investigation of these mechanisms.

## Conclusions

Sodium regulation is central to neuronal function and may be disturbed in patients with bipolar disorder. Monensin is an ionophore, meaning that it incorporates itself into the membrane and allows sodium to enter independent of cellular membrane proteins. While the mechanism remains obscure, the observation that the NMDA receptor antagonist, AP5, normalizes [Na]_i_ only in olfactory neuroepithelial precursors obtained from individuals with bipolar illness suggests that the NMDA receptor may play an important role in the ion dysregulation of excitable tissues of people with bipolar illness.

## Data Availability

All the data for this project are presented in this paper.

## Abbreviations

AP5: D-2-amino-5-phosphonopentanoate
BP: Bipolar disorder
MEM: Minimum essential media
NMDA: N-methyl-D-aspartate
[Na]_i_: Intracellular sodium concentration
ONP: Olfactory neuroepithelial precursor
